# Supplementary material for: Effective killing of Mycobacterium abscessus biofilm by nanoemulsion delivery of plant phytochemicals
Source: Microbiol Spectr. 2025 Jan 28;13(3):e02166-24. doi: 10.1128/spectrum.02166-24 (PMC11878076; doi:10.1128/spectrum.02166-24)
Supplement: Supplemental material — Fig. S1 and S2. [file spectrum.02166-24-s0001.pdf]

Supplemental Material for

Effective killing of *Mycobacterium abscessus* biofilm by nanoemulsion delivery of plant phytochemicals

Casey Albano [a], Ahmed Nabawy [b], Wyatt C. Tran [a,], Malavika Prithviraj [a], Takehiro Kado [a], Muhammad Aamir Hassan [b], Jessa Marie V. Makabenta [b], Vincent M. Rotello [b], and Yasu S. Morita [a,*]

a Department of Microbiology, University of Massachusetts, Amherst, MA 01003
b Department of Chemistry, University of Massachusetts, Amherst, MA 01003

* ymorita@umass.edu

This PDF file includes:

Figures S1 and S2

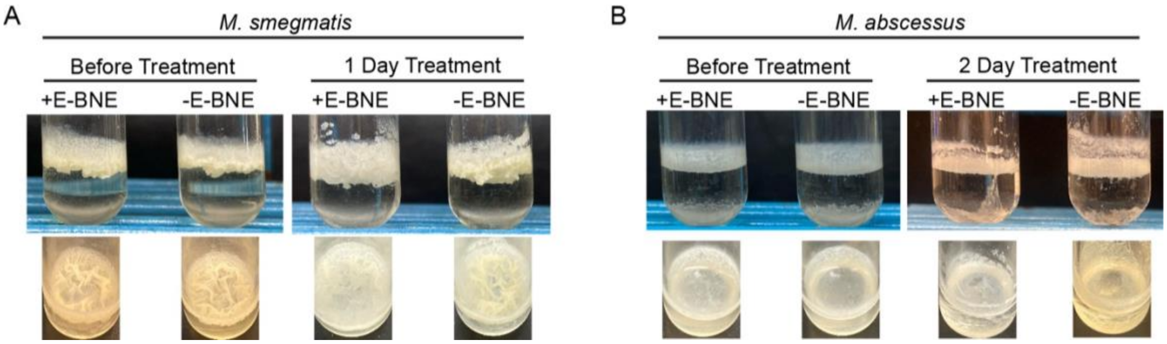

Figure S1. Pellicle growth of *M. smegmatis* and *M. abscessus*. A) *M. smegmatis* was grown in M63 broth for 5 days (before treatment). Medium was replaced with a fresh M63 containing E-BNE as described in Materials and Methods and incubated for 1 day (1 Day Treatment). **B)** *M. abscessus* was grown in M63 broth for 7 days (before treatment). Medium was replaced with a fresh M63 containing E-BNE and incubated for 2 days (2 Day Treatment). Pellicle was disrupted when medium was replaced to initiate the BNE treatment, but BNE treatments did not affect the overall appearance of the pellicles significantly.

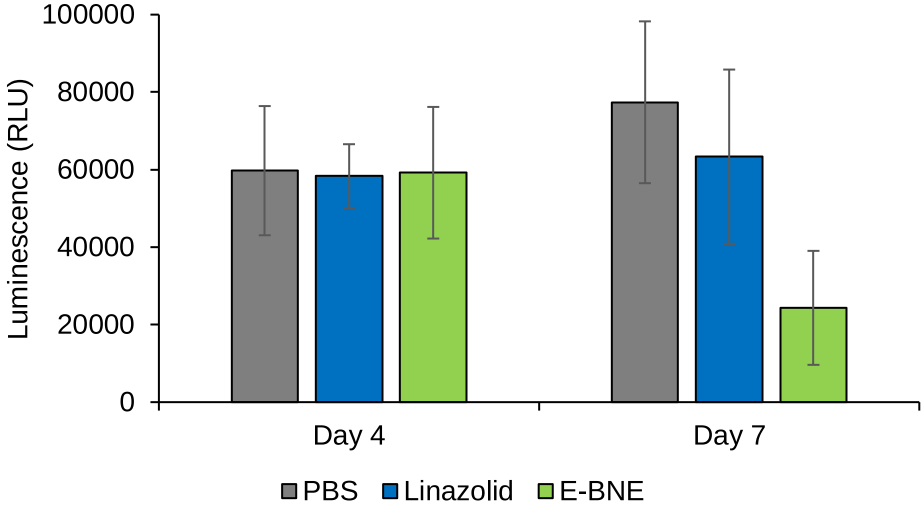

Figure S2. Luminance integrated density analysis of luciferase-expressing *M. abscessus*. Infected wound images obtained from IVIS before (day 4) and after treatment (day 7) of PBS, linezolid and E-BNE were quantified.
